# Preparation and Electrochemical Characterization of Organic–Inorganic Hybrid Poly(Vinylidene Fluoride)-SiO_2_ Cation-Exchange Membranes by the Sol-Gel Method Using 3-Mercapto-Propyl-Triethoxyl-Silane

**DOI:** 10.3390/ma12193265

**Published:** 2019-10-07

**Authors:** Yanhong Li, Zhiwei Li, Yanjuan Li, Wenxue Guan, Yangyang Zheng, Xuemin Zhang, Sanfan Wang

**Affiliations:** 1 School of Environmental and Municipal Engineering, Lanzhou Jiaotong University, No.88, Anning West Road, Lanzhou 730070, China; sxlizhiweiq@163.com (Z.L.); mmlyj2003@163.com (Y.L.); gwx1032827598@163.com (W.G.); zhengyy8018@163.com (Y.Z.); m13919848281@163.com (X.Z.); sfwang1612@163.com (S.W.); 2 Engineering Research Center of Water Resources Utilization in Cold and Drought Region, Ministry of Education, School of Environmental and Municipal Engineering, Lanzhou Jiaotong University, No. 88, Anning West Road, Lanzhou 730070, China

**Keywords:** cation-exchange membrane, polyvinylidene fluoride, oxidative stability, membrane resistance

## Abstract

A new synthesis method for organic–inorganic hybrid Poly(vinylidene fluoride)-SiO_2_ cation-change membranes (CEMs) is proposed. This method involves mixing tetraethyl orthosilicate (TEOS) and 3-mercapto-propyl-triethoxy-silane (MPTES) into a polyvinylidene fluoride (PVDF) sol-gel solution. The resulting slurry was used to prepare films, which were immersed in 0.01 M HCl, which caused hydrolysis and polycondensation between the MPTES and TEOS. The resulting Si-O-Si polymers chains intertwined and/or penetrated the PVDF skeleton, significantly improving the mechanical strength of the resulting hybrid PVDF-SiO_2_ CEMs. The -SH functional groups of MPTES oxidized to-SO_3_H, which contributed to the excellent permeability of these CEMs. The surface morphology, hybrid structure, oxidative stability, and physicochemical properties (IEC, water uptake, membrane resistance, membrane potential, transport number, and selective permittivity) of the CEMs obtained in this work were characterized using scanning electron microscope and Fourier transform infrared spectroscopy, as well as electrochemical testing. Tests to analyze the oxidative stability, water uptake, membrane potential, and selective permeability were also performed. Our organic–inorganic hybrid PVDF-SiO_2_ CEMs demonstrated higher oxidative stability and lower resistance than commercial Ionsep-HC-C membranes with a hydrocarbon structure. Thus, the synthesis method described in this work is very promising for the production of very efficient CEMs. In addition, the physical and electrochemical properties of the PVDF-SiO_2_ CEMs are comparable to the Ionsep-HC-C membranes. The electrolysis of the concentrated CoCl_2_ solution performed using PVDF-SiO_2_-6 and Ionsep-HC-C CEMs showed that at the same current density, Co^2+^ production, and current efficiency of the PVDF-SiO_2_-6 CEM membrane were slightly higher than those obtained using the Ionsep-HC-C membrane. Therefore, our novel membrane might be suitable for the recovery of cobalt from concentrated CoCl_2_ solutions.

## 1. Introduction

**Figure 1:**
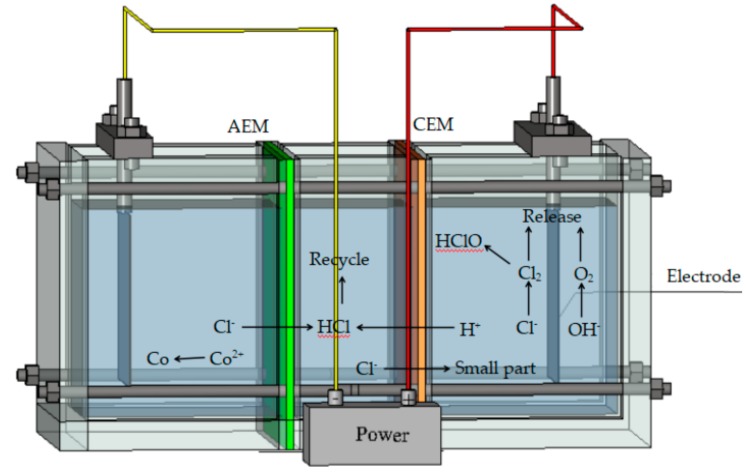
Schematic of electrolysis unit for “double membrane three chamber” cobalt recovery process (CEM is cation exchange membrane; AEM is anion exchange membrane).

Ion exchange membranes (IEMs) are widely used for industrial separation, energy generation, and water desalination [1,2,3,4,5,6,7,8,9]. The ion perm-selectivity between co-ions and counter-ions, which is the main property of IEMs, makes all these industrial applications possible and efficient. Such membranes belong to a class of dense polymeric membranes with charges present in the polymer matrix, which selectively permits counter-ions while blocking co-ions [10]. However, most membranes have a short time in which they can recover cobalt ions from concentrated CoCl_2_ solutions (see the schematics shown in Figure 1), mainly because the cation exchange membranes (CEMs) cannot completely block Cl^−^ during electrolysis. Some Cl^−^ still passes through CEMs and forms Cl_2_ on the anode, which then reacts with the water forming HClO, which is a strong oxidant. During the electrolysis of the concentrated CoCl_2_ solutions, the CEMs can easily be damaged by HClO (formed from Cl_2_ generated at the anode) and by OH and HO_2_·radicals (formed from incomplete reduction of oxygen diffusing through the membranes) [11]. This damage might severely affect the ion-exchange point of the membrane, which might lead to lower IEC values and, as a result, poor CEM performance. The oxidative degradation of the IEMs could start from the neutralization of -SO_3_H, which is the main functional groups providing the ion exchange capacity for CEMs [12]. It was shown that even small amounts of Cl_2_ can deteriorate IEMs by irreversibly decreasing their long-term perm-selectivity [13,14,15,16]. Thus, the perm-selectivity or oxidative stability of CEMs need to be improved for their membranes to be usable for the “double membranes three-chamber” cobalt recovery process. 

The service life of the CEMs used for the treatment of the concentrated CoCl_2_ solutions can be improved using several different methods. One method involves the improvement of CEM perm-selectivity, which will decrease the Cl^−^ leakage rate and, as a result, reduce the amount of Cl_2_ generated at the anode [17]. Another method is based on the enhancement of the oxidative stability of the CEMs to withstand attacks from the aggressive HClO, which will help to prolong the membrane’s service life. Thus, to implement these processes, physically and chemically stable IEMs with high perm-selectivity or good oxidative stability are needed. Nafion is physically and chemically stable and can be used to treat concentrated CoCl_2_ electrolytes. However, its industrial applications are limited because of its high cost [18,19]. The Ionsep-HC-C membrane is more reasonably priced and, at the same time, meets the requirements for the treatment of the concentrated CoCl_2_ electrolytes [20]. However, oxidation stability of Ionsep-HC-C membrane is poor. One of the possible solutions to solve all these drawbacks is preparation of organic–inorganic hybrid composites, which, in fact, attracted considerable attention [21]. Because such hybrid membranes combine the properties of both inorganic compounds and organic polymers, they possess excellent mechanical, electrical, and optical properties [22]. Another very important advantage of these hybrid membranes is their reasonable cost, which is below the cost of the Nafion membranes.

Organic–inorganic hybrid silica-based IEMs are often one of the first choices for complex solution separation processes in regular or harsh conditions because of their high thermal stability, chemical inertness, and excellent separation performance [23,24]. Several methods for the preparation of organic–inorganic hybrid silica IEMs have been reported in the literature [25,26,27,28,29,30,31,32]. The first one involves impregnation of the sol containing silica-immobilized phosphotungstic acid (Si-PWA) particles onto porous PVDF film. Pandey et al. [25,26] prepared IEMs based on PVDF-supported Si-PWA and used it for treatment of direct methanol fuel. The thermal and oxidative stability of this novel membrane was much better than that of Nafion-117. The same group prepared IEMs consisting of Si-PWA and polyvinyl-alcohol (PVA) [27]. The tensile strength of the resulting membranes was 93 MPa, which is higher than the tensile strength of Nafion-117 (which is equal to 34 MPa). The ion-exchange capacity of the novel membrane was equal to 0.90 $\mathrm{meq}\cdot g^{-1}$, which is higher than capacity of other PVA-based membranes. Additionally, this membrane showed very good ion-selectivity with a Na^+^ transport number equal to 92%. The second method involves blending SiO_2_ nanoparticles with the polymer matrix. IEMs fabricated using this method demonstrated excellent thermal stability, medium membrane conductivity, and improved selectivity, all of which made the resulting membrane very efficient for the electrical separation processes [28]. Zuo et al. [29] studied the properties of organic–inorganic hybrid silica-containing IEMs and reported excellent performance for IEMs containing 2.0 wt% of nano-SiO_2_ with very good water content, ion exchange capacity (IEC), selectivity, and moderate membrane conductivity. The last membrane preparation method is based on the sol-gel process performed using organo-alkoxy-silanes. Peng et al. [30] prepared polymeric membranes by adding γ-glycidyl-oxy-propyl-trimethoxy-silane into PVA by the sol-gel method. The resulting membrane was excellent for the pervaporation-based separation of cyclohexane mixtures because of its excellent combination of selectivity and permeability. The thermal stability of this novel hybrid membrane was even better than that of pure PVA. The performance of organic–inorganic hybrid IEMs based on PVA-SiO_2_ composites was studied in basic and acidic conditions [21]. The IEC, conductivity, and perm-selectivity of acid-catalyzed hybrid membranes were higher than those of the base-catalyzed hybrid membranes. The properties of acid-catalyzed hybrid membranes, which can be used for chlorine-alkali separation, were comparable to Nafion. Li et al. [31] studied how pendant groups affect the micropore and gas permeation of SiO_2_ IEMs, which showed low H_2_ permeability. They also determined that the size of the pendant group affected the final H_2_ permeability properties of these membranes. Mosa et al. [32] studied the IEMs prepared using 3-mercaptopropyl tri-methoxy-silane (MPTMS) and 3-glycidoxy-propyl trimetoxysilane (GMTMS). These membranes exhibited methanol permeability lower than that of Nafion. Thus, most of the literature studies involved an analysis of IEM performance. Research on the oxidative stability of hybrid IEMs is lacking. 

Therefore, in this work, we developed alternate type of organic–inorganic hybrid CEMs based on PVDF-SiO_2_ were prepared using the sol-gel method. In addition, ion exchange functional groups were introduced to this hybrid membrane by oxidation of its -SH functional groups. The membranes were synthesized in three steps. First, films consisting of a PVDF network containing organo-alkoxy-silane groups were fabricated using the sol-gel technique. The second step involved treatment of these films with 0.01 M HCl to form and then introduce a three-dimensional Si–O–Si network into the PVDF matrix. The formation of entangled and/or interpenetrated networks (with Si–O–Si and PVDF chains) was very beneficial to the oxidative and mechanical stability of the resulting PVDF-SiO_2_ hybrid membranes. The final step was the oxidation of the -SH groups of MPTES to cation-exchanging -SO_3_H groups by H_2_O_2_. Unlike similar methods reported in the literature [33], in which inorganic polymer was produced during membrane formation, our method involves incorporation of the inorganic polymer after membrane formation.

## 2. Experimental Section

### 2.1. Materials

**Figure 2:**
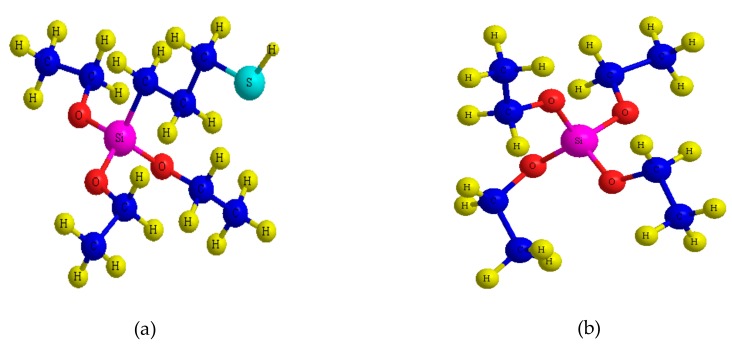
Molecular structures of the organo-alkoxy-silane: (**a**) 3-mercapto-propyl-triethoxyl-silane (MPTES); (**b**) tetraethyl orthosilicate (TEOS).

The Ionsep-HC-C membrane was obtained from Hangzhou EI Environmental Co. (Hangzhou, China). The Poly(vinylidene fluoride) (PVDF) with a molecular weight of ~1,000,000 g/mol was from Arkema (Paris, France). *N*,*N*-dimethylformamide (DMF) of analytical grade was purchased from Guanghua Reagent (Guangzhou, China). 3-mercapto-propyl-triethoxylsilane (MPTES) and tetraethyl orthosilicate (TEOS), the structures of which are shown in Figure 2, were supplied by Shandong West Asia Chemical Co. (Jinan, China) and used as received. Formaldehyde (CH_2_O), sodium hydroxide (NaOH), sulfuric acid (H_2_SO_4_), hydrochloric acid (HCl), and potassium chloride (KCl) were purchased from Guangdong Guanghua Technology Co. (Guangzhou, China). Anhydrous sodium sulfate (Na_2_SO_4_) (from Sinopharm Chemical Reagent Co., Shanghai, China) and hydrogen peroxide (H_2_O_2_) (30% w/v, from Tianjin Damao Chemical Reagent Co., Tianjin, China) were of AR grade. Distilled water was used throughout all experiments. 

### 2.2. Membrane Preparation

TEOS and MPTES were mixed at 1:2, 1:4, 1:6, 1:8, 1:10, and 1:12 molar ratios, with 55 mL of DMF. (After our preliminary tests, only membranes prepared with 1:4, 1:6, and 1:8 were selected for further detailed characterization). A total of 10 g of PVDF was added to each solution under constant stirring for 30 min. Then, the mixtures were ultrasonicated at room temperature for 2 h, after which they were placed in an oven heated to 60 °C for 3 h to remove air bubbles and form gels. The resulting gels were poured on a plexiglass plate, spread with a doctor blade, and allowed to dry at room temperature, after which the films were peeled off from the plexiglass plate and soaked in 0.01 M HCl, first for 2 h at 25 °C, and then for another 2 h at 60 °C [21]. Then, the membranes were immersed in room temperature H_2_O_2_ solution for 8 h, after which they were washed with water. All fabricated membranes were stored in 2 M NaCl solution. The as-fabricated membranes were stored in 2 M NaCl solution, with a final pH value of 2. The resulting membranes were marked as PVDF-SiO_2_-4, PVDF-SiO_2_-6, and PVDF-SiO_2_-8, depending on the silane ratio used. 

### 2.3. Characterization

#### 2.3.1. Morphology and Chemical Structure

Prior to the scanning electron microscopy (SEM) analysis, performed using UL TRA Plus instrument (Jena, Germany), the membranes were covered with a thin layer of gold deposited using EMS Q150 high vacuum ion sputtering instrument (Granbury, TX, USA), to decrease the sample charging. Prior to Au deposition, the membranes were washed with distilled water and then dried at 60 °C for 4 h. Regular fracture surfaces were prepared by soaking the membranes in liquid nitrogen and coating them with carbon under a vacuum. The top and fracture surface morphology of the membranes were then analyzed. Subsequently, SEM was performed on the different parts of the same membrane.

Fourier transform infrared spectroscopy (FTIR) spectra was performed using a VERTEX 70 spectrometer (Brooke, Switzerland) in the 4000–400 cm^−1^ range with a 1.5 cm^−1^ resolution. Prior to the test, the PVDF-SiO_2_ and Ionsep-HC-C membranes were soaked in a 2 M NaCl solution and then rinsed several times with distilled water and dried at 60 °C for 4 h. A small amount of the resulting powder was ground together with 100 mg of KBr using a mortar and then pressed into transparent pellets. Three pellets were prepared and tested for each membrane.

#### 2.3.2. Ion-Exchange Capacity and Water Uptake Measurements

The ion exchange capacity (IEC) of the organic–inorganic hybrid PVDF-SiO_2_ membranes, and the Ionsep-HC-C membrane was measured by acid-base titration. Before testing, each membrane was rinsed with distilled water, cut into three 1.5 cm × 1 cm pieces, and then placed in three different Erlenmeyer flasks. First, the membranes were exchanged for H^+^ by immersing them in 2 M H_2_SO_4_ for 24 h. Then, they were immersed in 2 M NaCl solution for another 24 h to exchange H^+^ for Na^+^. The exchanged solution was titrated with 0.05 M NaOH until an equivalent point (determined using phenolphthalein) was obtained. The volume of the NaOH consumed during titration was recorded three times. The IEC was calculated according to Equation (1). The final value was reported as an average of three measurements:

(1) $$IEC=\frac{nH^{+}}{W_{dry}}$$

where *IEC* is number of H^+^ per membrane weight (in mmol·g^−1^).

The test membranes were cut into three 2 cm × 2 cm pieces. Then, water uptake tests were performed at room temperature using weight changes before and after the membranes were submerged in distilled water for 24 h. The CEMs were removed from the distilled water, gently dabbed with tissue paper, and weighted immediately, after which the CEMs were dried at 60 °C until a constant weight was obtained. The water uptake (%) was calculated based on the formula reported elsewhere [26]. The final reported value was an average of the three measurements:

(2) $$Water\;uptake=\frac{\left(W_{wet}-W_{dry}\right)}{W_{dry}}\times 100$$

where *W_dry_* and *W_wet_* are the weights of the dry and wet CEMs, respectively.

#### 2.3.3. Membrane Area Resistance

**Figure 3:**
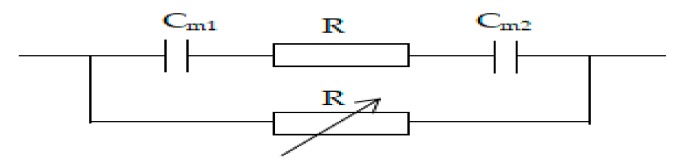
Schematic of an equivalent circuit diagram of the unit used to measure membrane resistance.

The area resistance of the organic–inorganic hybrid PVDF-SiO_2_ and Ionsep-HC-C membranes was tested in 2 M NaCl solution at 25 °C using an electrochemical workstation (PGSTAT128N, Metrohm, Beijing, China). This method can effectively eliminate the influence of concentration polarization and has high test precision and good reproducibility. Before the measurements, CEMs were washed with distilled water and then soaked in 2 M NaCl for over 24 h to maximize the CEM conversion into an Na^+^ type CEM, after which each CEM was placed in a two-compartment cell. 2 M NaCl was then added to both sides of the membrane for further membrane resistance measurements, during which the electrochemical workstation was connected to a membrane electrolysis unit. For simplicity, the ion-exchange membrane and solution resistance were assumed to be equal to their corresponding impedance values. At the same time, the electric double layer at the membrane/solution interface was assumed to be equal to the capacitive reactance (see Figure 3 for a graphical presentation of the corresponding equivalent circuit). The area resistance was calculated using Equation (3) [34]. Each resistance test used a 5 × 5 cm membrane but only 1 cm^2^ area was tested. Three different pieces of each membrane were tested. The final reported value was an average of the three independent measurements:

(3) $$R_{m}=[\frac{R_{1}R^{\prime }}{R^{\prime }-R_{1}}-\frac{R_{0}R^{\prime }}{R^{\prime }-R_{0}}]\times \frac{\pi D^{2}}{4}$$

where *R*_m_ is the membrane area resistance (in Ω·cm^2^); R_1_ is a sum of the resistances of the membrane and electrolyte solution (in Ω); *R*_0_ is the resistance of the electrolyte solution (in Ω); *R*’ is the resistance of the variable resistor (in Ω); and *D* is a cross-section diameter of the cell (in cm).

#### 2.3.4. Membrane Potential, Transport Number, and Selective Permittivity

Membrane potential is the sum of Donnan and the diffusion potential, which depends on ion distribution in the membrane pores and on the ion mobility of the membrane relative to the external phase [35,36,37,38,39,40]. This value was determined with reference to standard methods using equilibrated CEMs with solutions containing different KCl concentrations (C_1_ and C_2_, equal to 0.1 and 0.2 M, respectively) [41], which were placed on different sides of the membrane. The membrane’s actual potential was tested against 1 and 2 M KCl solutions. Prior to these measurements, the membranes were equilibrated in 1.5 M KCl solution for over 24 h. Solutions on both sides of the membrane were stirred continuously to reduce the boundary effect. 

The transmembrane potential was measured by connecting both membrane sides with a calomel electrode using KCl bridges. Management was performed using a digital automatic multimeter (DT-830-B, Digital Multimeter, Zhangzhou, China). Measurements were repeated until a constant membrane potential was obtained. The value of the first potential measurement was recorded for each membrane. Next, each membrane was cut into 2 pieces, and the potential measurements were repeated again. This was performed three times for each membrane with a fresh membrane piece each time. The final membrane potential value was the average of these three measurements. This E_m_ was then used to calculate the corresponding number of migrations and selected permeability according to Equations (4) and (5). After these data were subjected to error analysis, they were plotted:

(4) $$t_{+}=\frac{E_{m}}{2E_{0}}$$

where *E*_m_ and *E*_0_ are the measured and an ideal membrane potentials (in mv), respectively.

The ideal membrane potential (*E*_0_) was calculated using the following equation [42]:

(5) $$E_{0}=-(2{\bar{t}}_{+}-1)\frac{RT}{F}\ln\frac{a_{2}}{a_{1}}$$

where ${\bar{t}}_{+}$ is number of transported co-ions, R is the gas constant, T is the temperature, n is the electrovalence of co-ions, and $a_{1}$ and $a_{2}$ are the solution electrolyte activities at the corresponding contact membrane surfaces.

Selective permittivity is the most important IEM performance parameter reflecting the selective permeation ability of the membrane relative to the different ions and is typically expressed by the number of the migrating ions. The number of migrations is calculated as a percentage of the ions passing through the CEMs (see Equation (5)). It is often used to express the selectivity of the CEMs relative to certain co-ions. The selective permittivity of cations was calculated using the following Equation (6):

(6) $$P=\frac{{\bar{t}}_{+}-t}{1-t}$$

where t is transport number of the solution; t, which reflects the migration of the cations in the NaCl solution, is equal to 0.39 at 25 °C [34,35].

#### 2.3.5. Oxidative Stability

Fenton’s reagent, consisting of 3 wt% H_2_O_2_ and 3 ppm FeSO_4_, was used to determine the oxidative stability of PVDF-SiO_2_ and Ionsep-HC-C membranes. Four rectangular (1.5 cm × 2 cm) pieces of each membrane were dried at 60 °C and then weighed, after which each piece was immersed in 150 mL of freshly prepared Fenton’s reagent. Pieces were oxidized for 3, 6, 9 and 12 h at a certain temperature (40, 60, 70, and 80 °C), respectively, after which the membranes were taken out, dried at 60 °C, and then weighed again. Subsequently, the four membranes were individually cut into 32 pieces (1.5 cm × 2 cm in size) and prepared for oxidative stability measurement as described at the beginning of this paragraph. The average of the three weight losses for each membrane at a given temperature was calculated using the formula shown below:

(7) $$Oxidative\;Stibility(\%)=\frac{\left(W_{1}(g)-W_{2}(g)\right)}{W_{1}(g)}\times 100$$

where *W*_1_ and *W*_2_ are dry membrane weights before and after oxidation, respectively.

#### 2.3.6. Treatment and Recovery of the Concentrated CoCl_2_ Wastewater 

Each membrane was cut into three 200 mm × 200 mm pieces, each of which was then used for electrolytic experiments at different current densities. Three measurements were performed for each membrane. The resulting electrolytic test data were converted to the average cobalt recovery and current efficiency. The operation control parameters for the electrolysis of the CoCl_2_ solution performed using our membranes were as follows: 1) “double membrane three chamber” electrolysis, 2) 200 mm × 200 mm membrane area, 3) 125 mm spacing between electrodes, and 4) 190–220 A/cm^2^ and a 4.6–5.4V operating current density and operating voltage, respectively. Additional parameters were a 10 L volume for the circulating solution (which was 3.5%–4.2% HCl), a 45–55 °C solution temperature, and 40–80 g/L CoCl_2_ with pH values equal to 0.5–1.5 pH.

## 3. Results and Discussion

### 3.1. Membrane Preparation

**Figure 4:**
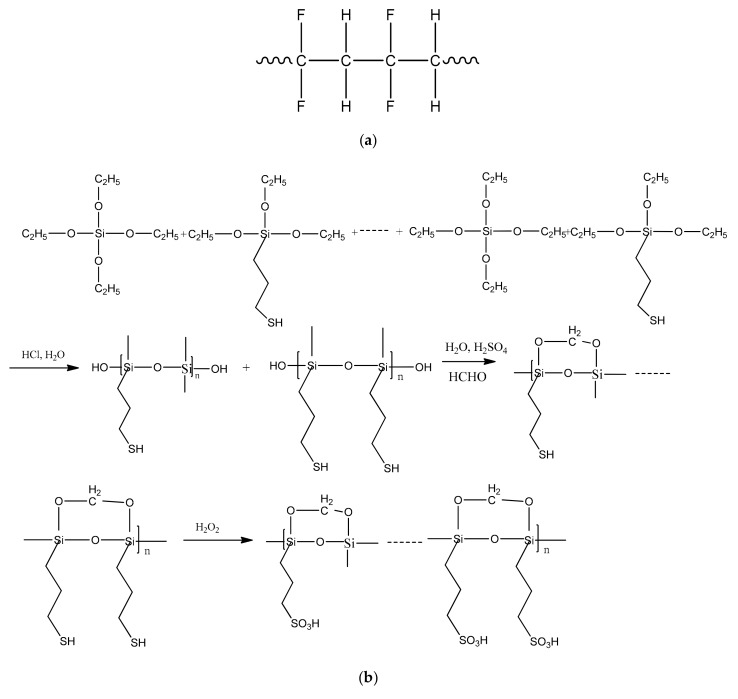
Schematics of the reactions occurring during the synthesis of organic–inorganic hybrid PVDF-SiO_2_ membranes: (**a**) polyvinylidene fluoride (PVDF) network chains; (**b**) Hydrolysis polycondensation of TEOS and MPTES.

TEOS and MPTEM were hydrolyzed and polymerized directly inside the film via catalysis with 0.01 M HCl. The resulting films were highly crosslinked in a solution [21] because freshly-formed Si–O–Si bonds were intertwined with the PVDF matrix. These crosslinks bonded not only with the polymer network but also with the silica parts. Covalent bonding between the polymer network and silica reinforced the interfacial interaction between the inorganic and organic phases. Thus, during this synthesis process, inorganic polymers formed after the film deposition. Interpenetration and/or entanglement between the Si–O–Si network and PVDF chains improved the mechanical properties and oxidative stability of the organic–inorganic hybrid PVDF-SiO_2_ CEMs. The corresponding reaction mechanism is displayed in Figure 4. 

Polycondensation of the alkoxy group of TEOS and MPTEM resulted in the formation of three-dimensional Si–O–Si linkages, which increased CEM oxidation stability. When used by itself, PVDF can be used to fabricate asymmetric membranes with very high quality [28]. Thus, a combination of additional Si–O–Si linkages and PVDF in a membrane can provide even better oxidative and thermal stability as well as good film-forming and mechanical properties [29]. 

### 3.2. CEM Morphology

**Figure 5:**
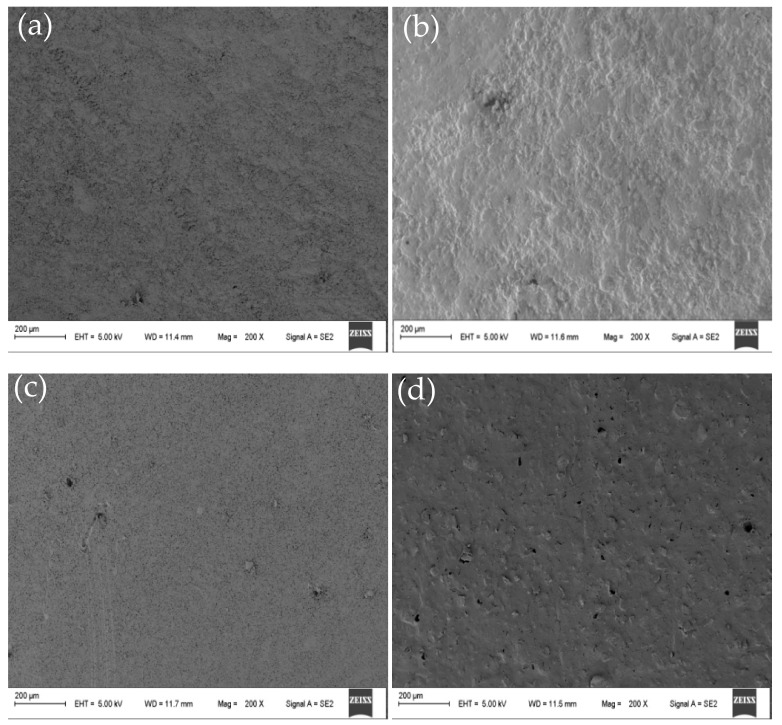
Representative SEM micrographs showing the surface morphology of the organic–inorganic hybrid PVDF-SiO_2_-4 (**a**), PVDF-SiO_2_-6 (**b**), PVDF-SiO_2_-8 (**c**), and Ionsep-HC-C (**d**) membranes.

**Figure 6:**
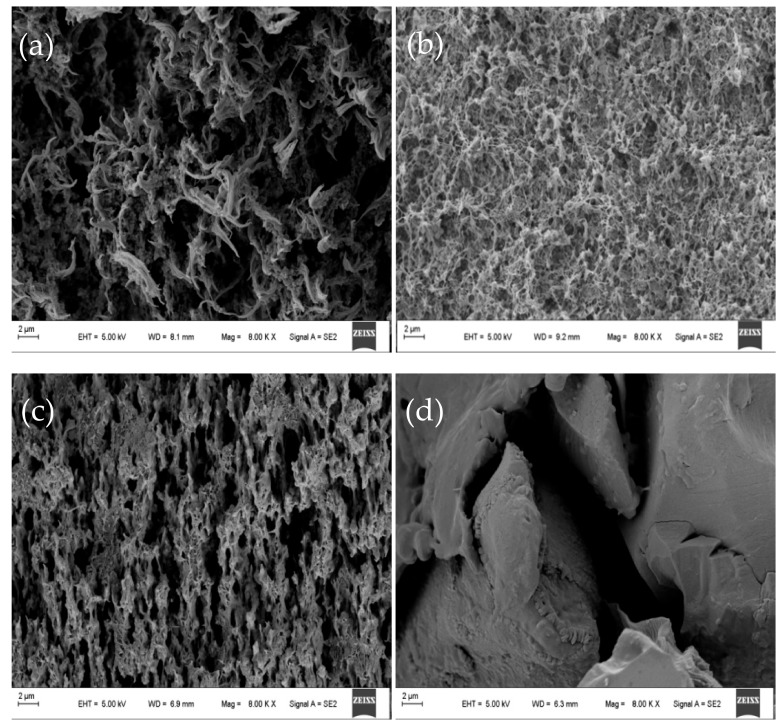
Representative SEM micrographs showing the cross-sections of the organic–inorganic hybrid PVDF-SiO_2_-4 (**a**), PVDF-SiO_2_-6 (**b**), PVDF-SiO_2_-8 (**c**), and Ionsep-HC-C (**d**) membranes.

The SEM showed that the structure of the membrane was uniform at different positions of the same membrane. SEM micrographs showing cross-sectional and surface morphology of the organic–inorganic hybrid PVDF-SiO_2_ membranes and Ionsep-HC-C membrane are shown in Figure 5 and Figure 6.

No visible pinholes and cracks were observed for our organic–inorganic hybrid PVDF-SiO_2_ CEMs (see Figure 5a–c), while the surface of the commercial Ionsep-HC-C membrane had clearly visible holes (see Figure 5d). Thus, our as-prepared membranes were very homogeneous with no detectable phase separation. The surface of the PVDF-SiO_2_-6 was rougher (see Figure 5b) than that of the PVDF-SiO_2_-4 (see Figure 5a) and of the PVDF-SiO_2_-8 (see Figure 5c) membranes, likely because of the formation of highly crosslinked networks. 

SEM analysis of a cross-section of the PVDF-SiO_2_-6 membrane showed significant porosity and a highly crosslinked network (see Figure 6b), especially when compared with PVDF-SiO_2_-4 (see Figure 6a) and PVDF-SiO_2_-8 (see Figure 6c) membranes. This may be due to the higher crosslinking density formed in the organic–inorganic network, since the formation of one network has a significant impact on the formation of another network (see Figure 5b) [27]. These voids and pores might increase the ξ potential of the PVDF-SiO_2_ membrane and improve its selective permeability. We also believe that such dense crosslinking contributed to the dimensional [43] and oxidative stability of the membranes. The Ionsep-HC-C membrane (see Figure 6d) has significant porosity (see Figure 5d), which might cause severe leakage of ions into the solution. SEM analysis was performed in different locations of the same membrane to confirm membrane uniformity and purity.

### 3.3. FTIR Analysis

**Figure 7:**
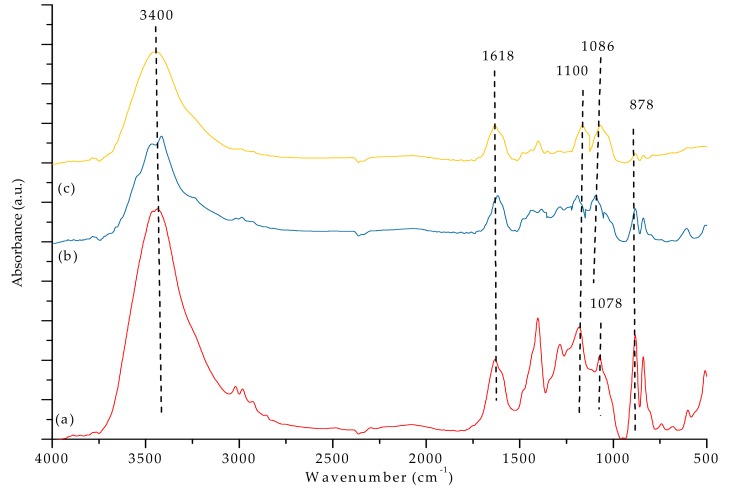
FTIR spectra of (**a**) PVDF/SiO_2_-4, (**b**) PVDF/SiO_2_-6, and (**c**) PVDF/SiO_2_-8.

**Figure 8:**
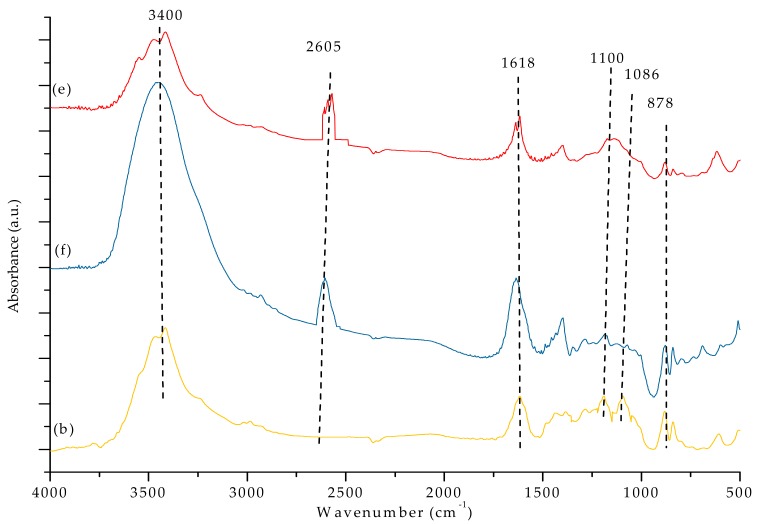
FTIR spectra of the PVDF-SiO_2_-6 membrane (**e**) before and (**f**) after cross-linking, as well as (**b**) after oxidation.

Figure 7a,b and Figure 8 show the FTIR spectra of our PVDF-SiO_2_ hybrid CEMs. The absorption bands associated with the -C-F functional groups were observed at 1100 cm^−1^ [44]. The peak at ~3400 cm^−1^ could be attributed to vibrations of -OH in the ≡SiOH group and/or remaining water (see Figure 7a–c and Figure 8e,f. Peaks at 1618 cm^−1^ very likely correspond to physically adsorbed water [21,32,45]. Absorption bands observed in the 2550–2600 cm^−1^ range (specifically at 2605 cm^−^^1^) are typical for the -S-H groups (see Figure 8e,f). The characteristic peaks of the -SO_3_H functional group were observed at 1086 and 1078 cm^−1^ (see Figure 7a–c) [21]. The -SH peak at 2605 cm^−1^ disappeared after oxidation, and the -SO_3_H peak appeared at 1086 cm^−1^ (see Figure 8). Meanwhile, the bands associated with the -SO_3_H peak appeared at 1086 and 1078 cm^−1^ (see Figure 7), indicating that -SH oxidized to -SO_3_H. The IEC values confirmed the presence of -SO_3_H groups (see discussion below). The characteristic peaks of the Si–O–C functional group (typically seen at ~1100 cm^−1^) overlapped with the adsorption bands corresponding to the -C-F group, which made them hard to distinguish [28]. Si-O-Si characteristic peaks appeared at 878 cm^−^^1^ (see Figure 7a–c and Figure 8e,f ).

### 3.4. Electrochemical Properties of CEMs

#### 3.4.1. Ion Exchange Capacity and Water Uptake

**Figure 9:**
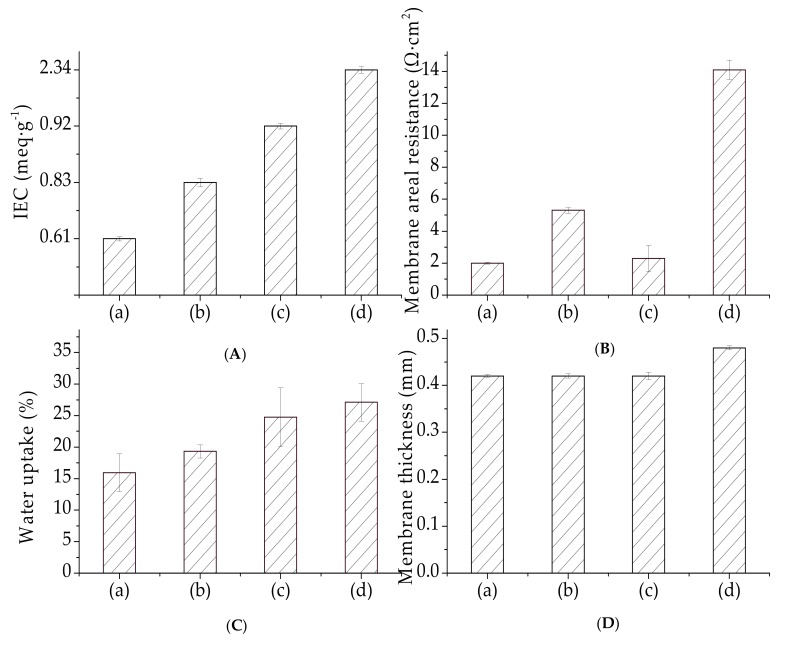
(**A**) IEC, (**B**) membrane resistance, (**C**) water uptake, and (**D**) membrane thickness for (**a**) PVDF/SiO_2_-4, (**b**) PVDF/SiO_2_-6, (**c**) PVDF/SiO_2_-8, and (**d**) Ionsep-HC-C membranes.

Ion exchange capacity (IEC) reflects the presence and amount of ion-exchange groups in a membrane [35]. The membrane IEC is important for water uptake because it depends on the density of the hydrophilic functional groups [46]. The IEC and water uptake values of our organic–inorganic hybrid PVDF-SiO_2_ and Ionsep-HC-C membranes are shown in Figure 9. 

IEC increased for the membranes prepared using higher amounts of MPTES. The IEC values for our membranes were in the 0.61 ± 0.05 to 0.92 ± 0.05 meq·g^−1^ range. These values are very similar to the values obtained for the PVA-based CEMs (equal to ~ 0.5-0.8 meq·g^−1^) reported in the literature [47,48]—somewhat lower than the IEC values of the Nafion membrane (equal to 0.996 meq·g^−1^) [21] and significantly lower than those of commercial Ionsep-HC-C membranes (2.34 meq·g^−1^). The IEC values of our organic–inorganic hybrid PVDF-SiO_2_ membranes were lower than the IEC values of the Ionsep-HC-C membrane, very likely because of the lower degree of oxidation of -SH to -SO_3_H or because of the crosslinked network formation [49,50]. High crosslinking density decreased the chain mobility and suppressed the swelling of the organic polymer network. The denser network of our organic–inorganic hybrid PVDF-SiO_2_ membranes might lead to smaller amounts and sizes of the H^+^ channels participating in ion-exchange and water absorption [43,51]. Thus, the H^+^ exchanged for Na^+^ during the acid–base titration, which showed low IEC numbers, did not reflect the true nature of our membranes. 

Water uptake affects the conductivity of the ion exchange membrane, because it is usually proportional to the concentration of -SO_3_H groups in dry membranes or IECs. Typically, water uptake increases as IEC increases because the mobility of hydronium ions in water enhances water uptake by the membrane. Water uptake obtained in this work followed a trend similar to the IEM (see Figure 9). Water uptake of the PVDF-SiO_2_ membranes was lower than the water uptake of the Ionsep-HC-C membrane. Thus, our highly crosslinked membranes in the electrolyte were more stable [43] than the sizes of the Ionsep-HC-C membrane. Therefore, the PVDF-SiO_2_ membranes are suitable for long-term use in applications related to the electrolysis of concentrated CoCl_2_ solutions.

#### 3.4.2. Electrical Resistance

The area resistance of our PVDF-SiO_2_ membranes in comparison to the Ionsep-HC-C membrane is shown in Figure 9. Knowing the membrane area resistance is important to assess the contribution of different functional groups [52]. Experimental studies reported in the literature showed that the resistance of IEMs is affected by the membrane structure, the relative sizes of the mobile phases, and the electrostatic interactions between the membrane charges and mobile cations [53,54,55]. 

The area resistance of the PVDF/SiO_2_-6 membrane was the highest out of all the membranes prepared in this work, very likely because of the existence of narrow ion transfer pathways (shown in Figure 6a–c) in the membrane matrix (see Figure 9). The area resistance of the organic–inorganic hybrid PVDF/SiO_2_ CEMs was higher than that of the Nafion-117 membrane [22] but lower than that of the Ionsep-HC-C membrane (very likely because of the lower thickness of our CEMs). Therefore, the utilization of PVDF-SiO_2_ membranes for the electrolysis of concentrated CoCl_2_ solutions is more economical than that of the Ionsep-HC-C membranes. The standard deviation values for the IEC, water absorption, and membrane resistance values are shown in Figure 9. All of these values are <10%, indicating good physical membrane properties.

#### 3.4.3. Membrane Potential, Transport Number, and Selective Permittivity

When membranes are used to separate unequal concentrations of electrolytes, an electrical potential is generated across the membrane because of the different mobility of co-ions and counter-ions. The magnitude of the membrane potential depends on the electrical characteristics of the membrane and the characteristics and concentration of the electrolyte [56]. 

**Figure 10:**
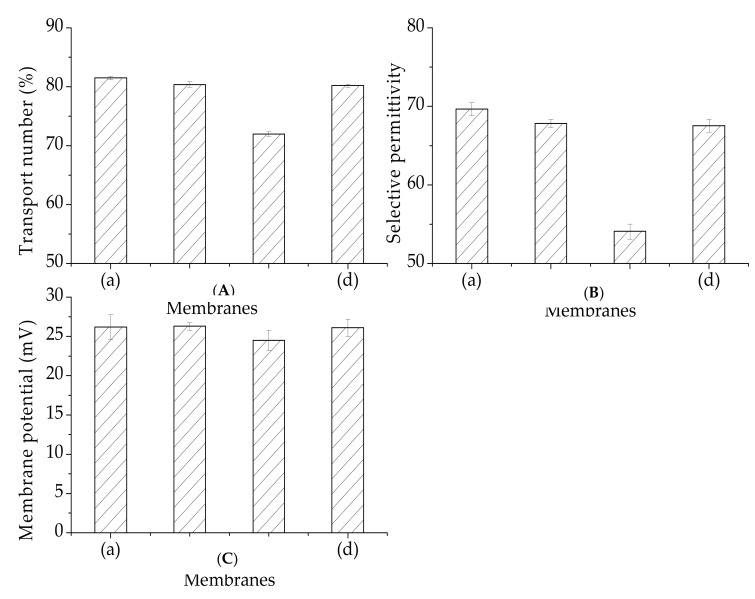
(**A**) Membrane potential, (**B**) transport number, and (**C**) permselectivity for (**a**) PVDF-SiO_2_-4, (**b**) PVDF-SiO_2_-6, (**c**) PVDF-SiO_2_-8, and (**d**) Ionsep-HC-C membranes.

As seen in Figure 10, the transport numbers and selective permittivity values for the membranes (PVDF-SiO_2_-4 and PVDF-SiO_2_-6) prepared in this work were similar to those of the Ionsep-HC-C membrane. The transport number and selective permittivity of the PVDF-SiO_2_-8 membrane was the lowest. The membrane potential and selective permittivity values followed the same trend as the transport numbers. The effect of this selective permittivity may be attributed to the fixed density of the change and to the pore density [28]. The fixed change density strengthened the Donnan exclusion of co-ions. Thus, PVDF-SiO_2_-8 very likely has relatively large pores, which might lead to ion leakage. This assumption was confirmed by our IEC and water uptake test results. It can be seen from Figure 10 that the standard deviations of the membrane potential, migration number, and selective permeability are small, indicating stable physicochemical membrane properties.

### 3.5. Oxidative Stability

**Figure 11:**
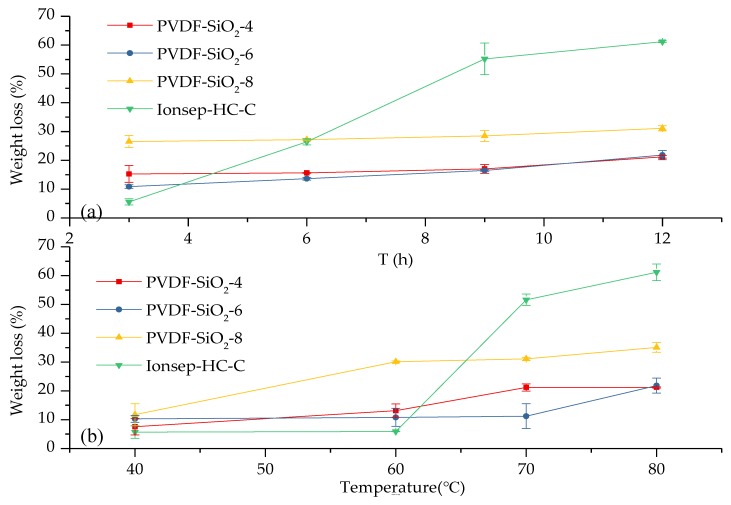
Weight loss of the organic–inorganic hybrid PVDF-SiO_2_ and Ionsep-HC-C membranes as a function of the temperature of the Fenton’s reagent solution during (**a**) 3, 6, 9, and 12 h tests at 80 °C and (**b**) during 12 h tests at 40, 60, 70, and 80 °C.

The 3 h weight loss for the Ionsep-HC-C membrane (which was equal to 5.6%) indicates that this membrane had the best oxidation stability out of all the membranes tested in this work (see Figure 11a). The weight loss of our PVDF-SiO_2_ membranes was >5.6%, very likely because of the following reasons. First, the aldol reaction was incomplete during membrane preparation, and ≡SiOH was not completely converted to Si–O–Si with a three-dimensional stable structure, which resulted in a relatively high weight loss during the first three hours. Second, the weight loss of our membranes was primarily due to the partial damage of the PVDF skeleton, which prevented PVDF matrix from forming complete chains. The 6–12 h weight loss of the Ionsep-HC-C membrane increased significantly, while the weight losses of the PVDF-SiO_2_ membranes barely changed as oxidation time increased. Thus, the oxidation stability of the membranes synthesized in this work was better than that of the Ionsep-HC-C membrane. The order of the oxidation stability of the membranes, from strongest to weakest, is WPVDF-SiO_2_-6 > PVDF-SiO_2_-4 > PVDF-SiO_2_-8 > Ionsep-HC-C.

As seen in Figure 11b, the weight loss of the Ionsep-HC-C membrane at 40 and 60 °C was lower than that of the PVDF-SiO_2_ membranes. The weight loss of the Ionsep-HC-C membrane significantly increased as the oxidation temperature increased, while the weight loss of the PVDF-SiO_2_ membranes were lower at higher temperatures (70 and 80 °C). The reason for such behavior is the damage to the Ionsep-HC-C membrane at high temperatures, which indicates its poor oxidation stability at elevated temperatures. Another reason might be the higher IEC values and more ion-exchange functional groups available in the Ionsep-HC-C membrane.

Our experimental results demonstrated that crosslinked silica enhanced the oxidative stability of the CEMs, likely because of the following factors. First, crosslinked silica increased the polymer chain’s density, which increased IEM’s resistance to radical attacks and HClO. Second, crosslinked silica improved the polymer’s strength and quality. Polymer chains damaged by radicals and HClO might still remain attached to the polymer network via the crosslinking site. Thus, the complete damage of the polymer by the radicals and HClO will take longer. Thus, the three-dimensional Si–O–Si skeleton of the PVDF-SiO_2_ membranes significantly improved their oxidation resistance. Figure 11 shows that the standard deviations of the weight loss values of the PVDF-SiO_2_-4, PVDF-SiO_2_-6, PVDF-SiO_2_-8, and Ionsep-HC-C membranes were small, which indicates that the PVDF-SiO_2_ membranes had good oxidation stability and are superior to the Ionsep-HC-C membrane. It also shows that the oxidation stability of PVDF-SiO_2_-6 was the best among the four membranes prepared in this work.

### 3.6. Treatment and Recovery of a High Concentration of Cobalt-Containing Wastewater

**Figure 12:**
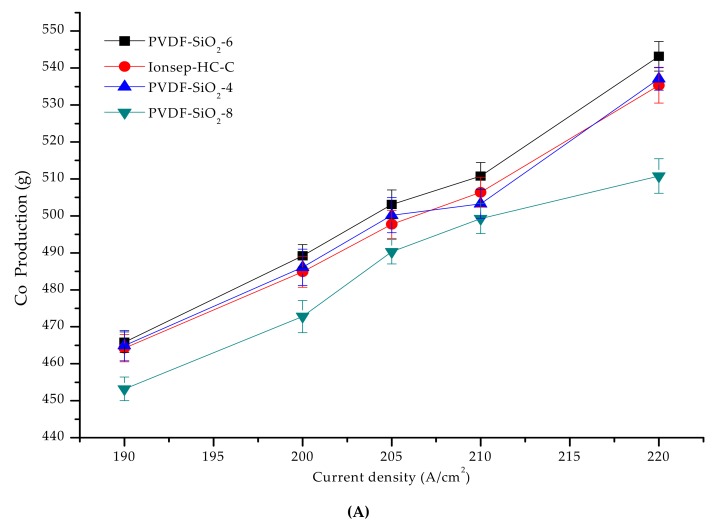

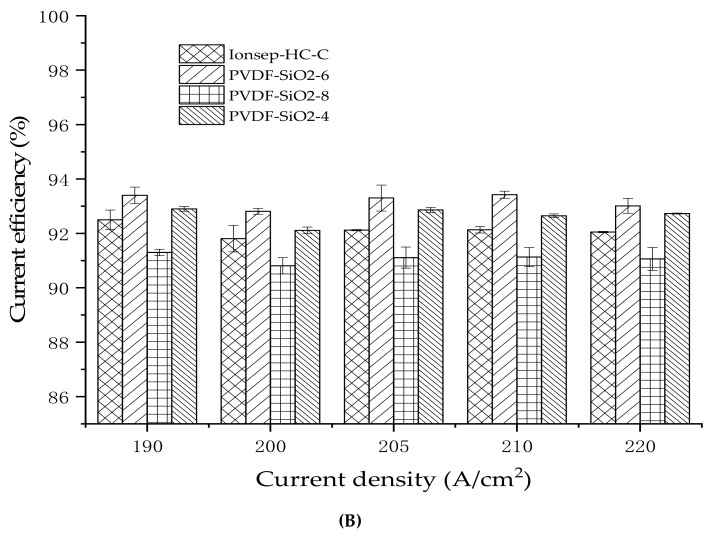
(**A**) Graph of the co-production of the four membranes as a function of the current density; (**B**) The relationship between the current efficiency and current density in the cobalt recovery process.

The current efficiency of the PVDF-SiO_2_ and Ionsep-HC-C membranes was high (above 91%) at 190–220 A/cm^2^ current density (see Figure 12B). As the current density increased, the order of cobalt production of the four membranes from high to low was PVDF-SiO_2_-6 > PVDF-SiO_2_-4 > Ionsep-HC-C > PVDF-SiO_2_-8, mainly because of the somewhat better selective permittivity of the PVDF-SiO_2_-6 membrane. Higher selective permittivity results in higher Co^2+^ production because each membrane cell automatically maintained the process’s neutrality. Because of the improved selective permittivity of the anode membrane, the percentage of H^+^ passing through the anode membrane from the anode chamber increased, while the amount of Cl^−^ entering the anode chamber through the anode membrane decreased. To maintain the electroneutrality of the middle compartment, it was necessary to increase the amount of Cl^−^ entering the intermediate compartment without changing the anion nature of the exchange membrane. Similarly, the amount of H^+^ passing through the anion exchange membrane decreased. Thus, the percentage of H^+^ at the cathode chamber (reduced to H_2_ at the cathode) decreased. At the same time, the production of Co^2+^ and current efficiency increased. Figure 12 shows that the standard deviation of the current efficiency and cobalt production is small, further indicating that our PVDF-SiO_2_ membranes are suitable for the recovery of highly concentrated CoCl_2_ solutions. 

## 4. Conclusions

Organic–inorganic PVDF-SiO_2_ hybrid CEMs were prepared by the sol-gel method, during which MPTES and TEOS were added to the PVDF slurry. The initial amount of the MPTES significantly affected the physicochemical and electrochemical properties of the resulting organic–inorganic PVDF-SiO_2_ hybrid CEMs. -SO_3_H functional groups were introduced to the membrane via the oxidation of existing -SH functional groups by H_2_O_2_. We used a novel method to prepare interlinked PVDF-based polymer membranes: inorganic Si–O–Si polymers were created when freshly-prepared films were soaked in HCl. 

The results of the antioxidative and membrane resistance tests revealed the adequate oxidative stability and low energy consumption of the membranes, which is essential for the fabrication of stable ion exchange membranes with a long lifetime. 

The electrochemical properties of our PVDF-SiO_2_ hybrid CEMs were compared with a commercial Ionsep-HC-C CEM. The oxidative stability of the membranes prepared in this work was better than that of the Ionsep-HC-C membrane, but its resistance was lower. Out of all membranes prepared in this work, the overall best performance was observed for the PVDF-SiO_2_-6 membrane. The PVDF-SiO_2_-6 and Ionsep-HC-C membranes were applied to the electrolysis of the concentrated CoCl_2_ solution. At the same current density and current efficiency, the cobalt recovery demonstrated by the PVDF-SiO_2_-6 membrane was slightly higher than that of the Ionsep-HC-C membrane. Thus, our novel membrane might be suitable for applications related to the electrolysis of concentrated CoCl_2_ solutions.
